# Association between hand grip strength and quality of life in children with cerebral palsy: a cross-sectional study

**DOI:** 10.7717/peerj.18679

**Published:** 2024-12-16

**Authors:** Mshari Alghadier, Nada Almasoud, Dalia Alharthi, Omar Alrashdi, Reem Albesher

**Affiliations:** 1 Department of Health and Rehabilitation Sciences, Prince Sattam bin Abdulaziz University, Kharj, Saudi Arabia; 2 Department of Physical Therapy, Maternity and Children’s Hospital in Alkharj, Kharj, Saudi Arabia; 3 Department of Physical Therapy, Alhada Armed Forces Hospital, Taif, Saudi Arabia; 4 Department of Physical Therapy, King Khalid Hospital, Hail, Saudi Arabia; 5 Department of Rehabilitation Sciences, Princess Nourah bint Abdulrahman University, Riyadh, Saudi Arabia

**Keywords:** Cerebral palsy, Hand grip strength, Quality of life, Association, HRQoL

## Abstract

**Background:**

Cerebral palsy (CP) covers a wide range of causes and symptoms. It is characterized by persistent motor and postural dysfunction caused by a non-progressing pathological lesion of the immature brain. Development of fine motor skills, such as the ability to manipulate objects with smaller muscles, is crucial for a child’s development. It is evident that there is a lack of hand grip strength (HGS) and quality of life (QoL) data in children with CP compared to typically developed (TD) children. Understanding the relationship between these factors might help facilitate healthcare provision and provide insight into rehabilitation programs. The aim of this study is to investigate the relationship between HGS and health-related quality of life (HRQoL) in children with CP compared to TD children.

**Methods:**

An experimental cross-sectional study was conducted and 60 children (30 CP and 30 TD) were chosen; age, gender, height, weight, body mass index, preferred hand, number of siblings, school attendance, and housing type data were collected. HGS was measured using a standard hand dynamometer, and HRQoL was measured using the KIDSCREEN-10 item questionnaire.

**Results:**

There was a statistically significant main effect of gender on the average HGS, *F* (1, 56) = 24.09, *p* < 0.001, and the KIDSCREEN-10 sum score, *F* (1, 56) = 8.66, *p* < 0.001, and the main effect of group on the KIDSCREEN-10 sum score, *F* (1, 56) = 17.64, *p* < 0.001. A significant correlation between HGS and the KIDSCREEN-10 sum score in the CP group (*r* = 0.35, *p* = 0.03), and the TD group (*r* = 0.56, *p* = 0.001).

**Conclusion:**

HGS was lower in children with CP, and girls had significantly lower HGS compared to boys in both groups, CP and TD children. HRQoL was significantly lower in children with CP, with boys reporting higher HRQoL on the KIDSCREEN-10 questionnaire compared to girls. Our data showed that the higher the KIDSCREEN-10 sum score is, the stronger the HGS of children in both groups. The results of this study indicate that hand grip strength may significantly impact the QoL of children with CP. A correlation between HGS and HRQoL points to the importance of improving strength in children with CP through interventions and directed rehabilitation programs.

## Introduction

As an umbrella term, cerebral palsy (CP) describes a variety of etiologies and clinical manifestations. It is often described as a non-progressing pathological lesion of the immature brain that causes persistent postural and motor problems (Bax et al., 2005). In developed countries, CP is the most common neurological disorder associated with physical disability in children (Michael-Asalu et al., 2019). Many symptoms may accompany CP, including spastic paresis, ataxia, dyskinesia, impaired sensation, cognitive disorders, speech problems, visual disturbances, and epilepsy (Bax et al., 2005; Odding, Roebroeck & Stam, 2006). In recent years, several studies have indicated a decline in the prevalence of CP worldwide (Jonsson et al., 2019; Robertson et al., 2017; Touyama et al., 2016). Multiple studies have reported that the prevalence of CP in Saudi Arabia ranges from 0.41 to 2.3 per 1,000 live birth (Al-Jabri et al., 2022; Al Salloum et al., 2011).

The development of motor skills is crucial for children’s ability to perform daily tasks, improve their physical health, and develop their cognitive skills (Van der Fels et al., 2015; Bolger et al., 2021; Dapp, Gashaj & Roebers, 2021). In typically developing children (TD) aged 8 to 12 years, muscle strength, coordination, and postural control usually increase as the central nervous system (CNS) matures (Patel et al., 2020; Cumberworth et al., 2007). However, children with CP commonly suffer from compromised CNS function, resulting in varying degrees of muscle weakness, spasticity, and coordination impairment (Cappellini et al., 2020; Lorentzen et al., 2019; Paul et al., 2022). Consequently, a lack of isometric strength can greatly affect their ability to maintain posture and perform tasks requiring stability and endurance. Due to this, children with CP may have difficulty participating in activities that facilitate motor skill development, adversely impacting their physical and social well-being, as well as their quality of life (QoL).

Children with CP have increased muscle tone due to brain injury in addition to limited hand function, coordination, and balance (Paul et al., 2022; Vitrikas, Dalton & Breish, 2020). This is thought to be due to the increased amount of muscle fibers required to accomplish a given task compared to TD children. The accumulation of collagen in myofibers has been associated with decreased muscle flexibility, and the impairment of the neuromuscular junction may affect muscle contraction (Arnaud et al., 2021). As a result, children with CP may experience difficulty dressing, eating, or writing, requiring assistive devices or caregiver assistance to accomplish tasks that TD children can achieve independently.

A child’s fine motor skills, which include the ability to manipulate objects with their smaller muscles, are essential to their development. Fine motor skills allow children to develop manual abilities, which later contribute to various cognitive abilities, such as writing and drawing (Tóth, 2017). An assessment of hand grip strength (HGS) is used to determine physical functioning, maximum voluntary force, musculoskeletal fitness, and the relationship between arm, trunk, and leg strength (Ortega et al., 2015; Wong, 2016). In both, CP and TD children, the HGS can indicate the child’s overall muscle strength and physical wellness (Savva et al., 2014; Peolsson, Hedlund & Öberg, 2001). It is among the easiest and most cost-effective used measurements of physical and hand function in children with CP (Schreuders et al., 2003).

Health-related quality of life (HRQoL) refers to an individual’s perception and subjective evaluation of their health and well-being within the context of their culture (The WHOQOL Group, 1995). Quality of life instruments are increasingly used as outcome measures within several settings such as clinical research, population health surveys, and clinical practice for both adults and children. A 2008 review identified 30 generic and 64 disease-specific instruments available for use with children and adolescents (Solans et al., 2008). Children and adolescents with CP showed similar HRQoL scores to their age-matched TD children except on measures of social participation and motor function (Colver et al., 2015; Shikako-Thomas et al., 2012; Power et al., 2020; Dickinson et al., 2007). Several factors have been identified as predictive of adverse HRQoL, including pain, parenting stress, and psychological problems (Dickinson et al., 2007; Böling et al., 2013; Im, Cho & Kim, 2019; Rapp et al., 2017). Even though motor impairment negatively affected functioning and participation, it had a much smaller impact on psychological well-being (Mc Manus, Corcoran & Perry, 2008).

Previous research has indicated a negative relationship between HRQoL and HGS with age in adult and older adults’ population (Halaweh, 2020; Musalek & Kirchengast, 2017; Kang, Lim & Park, 2018). A recent study has indicated that higher HGS is associated with higher cognitive function score such as working memory, speed, attention, and self-control in adults with CP (Heyn et al., 2023). Performing cognitive functions is essential for learning, working, and managing daily activities that may impact HRQoL. However, little is known about the association between HRQoL and HGS for children with CP. In order to develop effective interventions and strategies to improve the general well-being of children with CP, it is essential to understand the relation between HGS and HRQoL. Therefore, the aim of this study is to investigate the relationship between HRQoL and HGS in children with CP compared to their TD counterparts. Our hypothesis was that children with CP will have lower HGS and HRQoL scores than their TD peers, along with a role for gender and age in these differences.

## Materials and methods

### Participants

**Figure 1:**
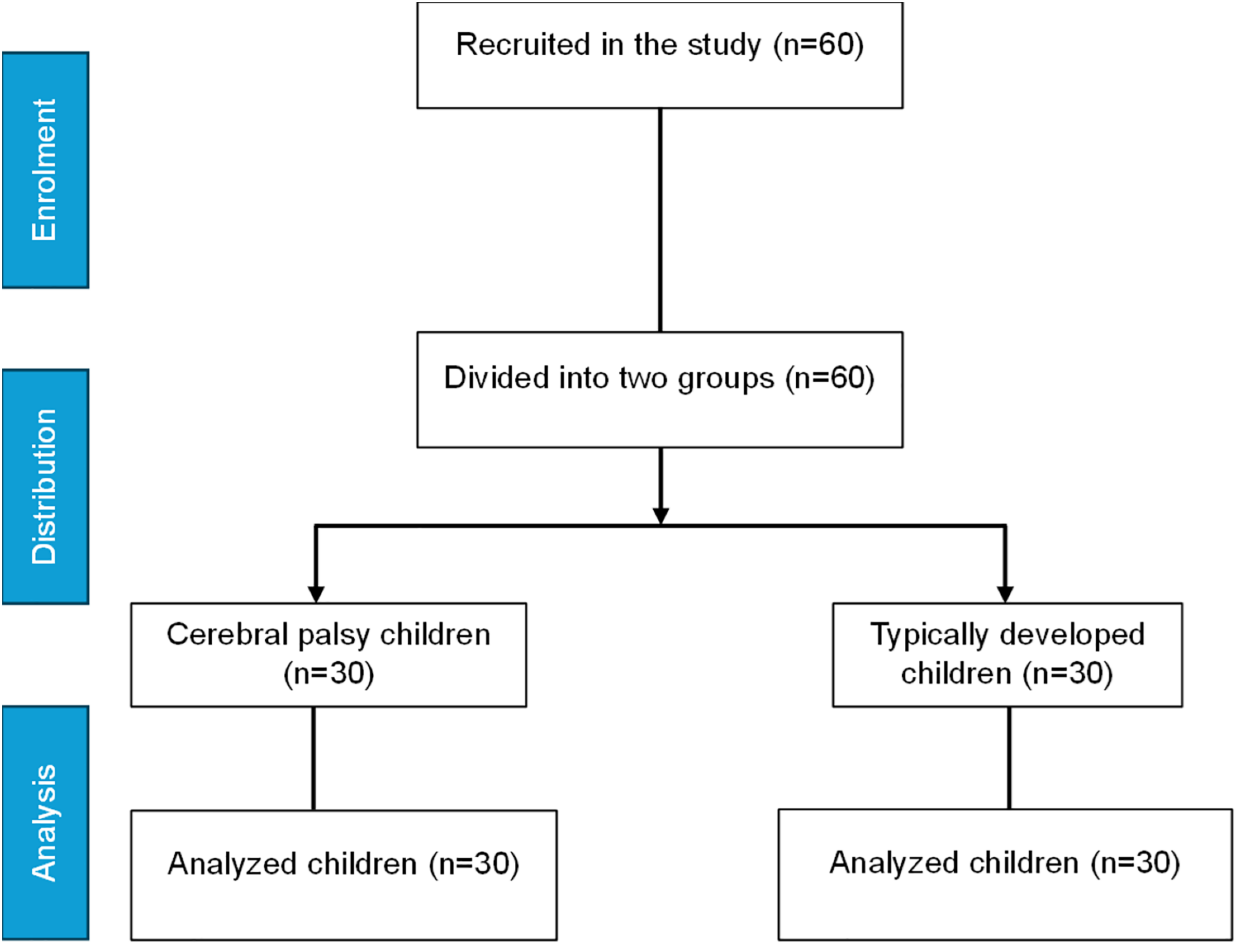
Study recruitment and distribution flow chart.

A total of 60 age-matched children (30 CP and 30 TD), aged 8–12 years, were recruited for this experimental cross sectional study through convivence sampling (Fig. 1). There were several eligibility criteria for CP children, including the ability to walk, use their upper limbs, Gross Motor Function Classification System (GMFCS) level I to III, and a sound cognitive ability. Cerebral palsy children with severe intellectual disabilities, unable to use upper limbs, GMFCS level IV and level V, or who have undergone surgery within the past six months were excluded from the study. There are five levels in the GMFCS, and it’s used to classify the gross motor function ability in children with cerebral palsy. In level I, the individual can walk without difficulty; however, gross motor skills are limited. In level II, the individual is able to walk without assistance; however, they are limited in their ability to walk outdoors and in public places. In level III, the individual can walk outside and in the community with the assistance of mobility devices. In level IV, children have limited mobility and are transported to and from the community and outdoors by power mobility. In level V, there are significant limitations in mobility, despite the use of assistive technology (Rosenbaum et al., 2002). Cerebral palsy patients were recruited from various healthcare facilities (clinics and hospitals) and TD children were recruited from different schools in Riyadh City, Saudi Arabia during the period of 1st of August 2023 to 1st of March 2024. The parents were informed of the goals, the procedures followed, and the objectives of the research study before a written informed consent was obtained.

### Procedure

During the visit, the participants had their demographic information collected; age, gender, height, weight, body mass index (BMI), preferred hand, number of siblings, school attendance, and housing type. The preferred hand was determined with an observation of activity of daily living performance such as writing, reaching, and grasping. Hand grip dynamometer was used to measure hand muscle strength, the Arabic versions of the GMFCS Family Questionnaire utilized to determine GMFCS level, and the Arabic version of the QoL KIDSCREEN-10 questionnaire used to examine the HRQoL. All outcome measures were collected by trained pediatric physiotherapist.

#### Hand grip strength

The American Society of Hand Therapists (ASHT) recommendations were followed to collect HGS by asking participants to sit on a chair facing the examiner with their feet flat on the ground. Their shoulder was neutrally rotated and adducted. The elbow was flexed at 90° with the forearm in neutral placement. The wrist was extended between 0° and 30° and ulnar deviated between 0° and 15° (American Society of Hand Therapists, 1981). Following verbal instructions and demonstration of the test position, three practice trials were performed alternating the preferred and non-preferred hand. Hand grip strength measured in kilograms (kg) using standard hand dynamometer (Baseline^®^ Hydraulic Hand Dynamometer, Fabrication Enterprises, White Plains, NY, USA). The concurrent validity and inter-instrument reliability of baseline hydraulic dynamometers are satisfactory, and it’s been validated and previously used to measure HGS in children with CP (Allen & Barnett, 2011; Gąsior et al., 2020; Mandanka & Diwan, 2020; Dekkers et al., 2020). Verbal encouragements (*i.e*., squeeze as hard as you can) were introduced and children performed three trials for the preferred hand, then the mean values of the three trials were recorded. Children were given one-minute to rest between trials to minimize the effects of fatigue (Trossman & Li, 1989).

#### KIDSCREEN-10

For children and adolescents, the KIDSCREEN QoL instrument is available in three versions: a 52-item version (Ravens-Sieberer et al., 2005, 2008), a 27-item version (Ravens-Sieberer et al., 2007; Robitail et al., 2007), and an index of 10 items (Ravens-Sieberer et al., 2010). The KIDSCREEN-10 questionnaire is a valid and reliable, fully open access tool used to evaluate the HRQoL of children aged from 8–18 (Ravens-Sieberer et al., 2010). Both a self-report and a parent-report versions are available, each containing 10 items scored on a 5-point scale. Multiple studies have used the KIDSCREEN-10 questionnaire for children with CP in different cultural contexts (Bingol et al., 2023; Braccialli et al., 2016; Maier et al., 2022; Milićević, 2023). The KIDSCREEN-10 items as reported by Ravens-Sieberer et al. (2010) are as follow: (1) Have you felt fit and well? (2) Have you felt full of energy? (3) Have you felt sad? (4) Have you felt lonely? (5) Have you had enough time for yourself? (6) Have you been able to do the things that you want to do in your free time? (7) Have your parent(s) treated you fairly? (8) Have you had fun with your friends? (9) Have you got on well at school? (10) Have you been able to pay attention? Answer categories item 1 and 9: not at all; slightly; moderately; very; and extremely. All other items: never; seldom; quite often; very often; and always.

Items 1 and 2 explore the level of the child’s/adolescent’s physical activity, energy and fitness. Items 3 and 4 cover how much the child/adolescent experiences depressive moods and emotions and stressful feelings. Items 5 and 6 ask about the child’s opportunities to structure and enjoy his/her social and leisure time and participation in social activities. Item 7 explores the quality of the interaction between child/adolescent and parent or carer and the child’s/adolescent’s feelings toward their parents/carers. Item 8 examines the nature of the child’s/adolescent’s relationships with other children/adolescents. Lastly, items 9 and 10 examine a child’s/adolescent’s perception of his/her cognitive ability and school performance. The responses were coded so that higher values were associated with better HRQoL; the sums were then calculated, and Rasch person parameters (PP) were assigned to each possible sum score. Based on the PPs, a mean of 50 and a standard deviation (SD) of approximately 10 were calculated (The KIDSCREEN Group Europe, 2006). Low scores indicate poor HRQOL, whereas high scores indicate better HRQOL.

### Statistical analysis

Descriptive statistics were used to characterize the sample; frequencies and percentages were reported for categorical variables, while mean and standard deviation (SD) were reported for continuous variables. The comparisons between groups were made according to gender and group (CP and TD). An independent sample t-test and Chi-square tests were performed to determine the differences in age, gender, height, weight, BMI, number of siblings, HGS, normalized HGS, preferred hand, housing type, and school attendance between groups. Normalized HGS was calculated by dividing the absolute average HGS by body weight. Two-way analysis of variance (ANOVA) tests were performed to analyze the differences in HGS and KIDSCREEN-10 sum scores between gender and group. A Kolmogorov–Smirnov test was used to test for normality on the KIDSCREEN-10, and a Mann–Whitney U test was conducted to evaluate the difference in KIDSCREEN-10 items between groups. The correlation between the KIDSCREEN-10 sum score and HGS was assessed using Spearman’s rank correlation coefficient. The strength of correlation was interpreted as recommended by Altman (1990); no correlation: 0 to ±0.3, low correlation: ±0.3 to ±0.5, moderate correlation: ±0.5 to ±0.7, high correlation: ±0.7 to ±0.9, very high correlation: ±0.9 to ±1.0, and complete correlation: ±1. Statistical significance was set at *p* < 0.05, and data were analyzed using R version 4.0.3 (R Core Team, 2020).

### Ethical considerations

Informed consent from the participant’s parents or legal guardians was acquired prior to data collection. The Declaration of Helsinki’s ethical guidelines were followed when conducting this investigation. The study was approved by the Departmental Ethical Committee, Department of Health and Rehabilitation Sciences, Prince Sattam bin Abdulaziz University, Saudi Arabia (No. RHPT/023/015). All methods were performed in accordance with relevant institutional review boards and regulations. Throughout the study, participant data confidentiality and anonymity were guaranteed.

## Results

A total sample of 30 CP patients and 30 TD children were included in the study. Weight, BMI, and number of siblings were statistically significantly different between CP and TD children; however, height and HGS were not statistically significant between the two groups (*p* = 0.08, *p* = 0.11, respectively). Table 1 summarizes the demographic characteristics of the sample divided by groups.

**Table 1:** Demographic characteristics of the sample included divided by two groups; cerebral palsy and typically developed children.

Variable	Typically developed	Cerebral palsy	*p*-value
Age, y	9.27 ± 1.41	9.53 ± 1.11	0.41
Gender (%)			
Boys	14 (23.3)	14 (23.3)	1.0
Girls	16 (26.7)	16 (26.7)	
Height, cm	127.63 ± 9.75	122.83 ± 11.12	0.08
Weight, kg	33.86 ± 10.49	22.78 ± 7.15	<0.001*
BMI	20.44 ± 4.41	14.81 ± 2.68	<0.001*
Number of siblings	3.87 ± 2.62	2.60 ± 1.25	0.02*
HGS 1, kg	18.80 ± 10.78	15.37 ± 5.89	0.13
HGS 2, kg	18.53 ± 10.10	15.37 ± 5.94	0.14
HGS 3, kg	19.27 ± 10.09	15.67 ± 5.64	0.09
Average HGS, kg	18.87 ± 10.27	15.47 ± 5.72	0.11
Normalized HGS, kg	0.14 ± 0.07	0.12 ± 0.04	0.25
Preferred hand *n* (%)			
Right	28 (46.7)	20 (33.3)	0.01*
Left	2 (3.3)	10 (16.7)	
Housing type (%)			
House with yard	20 (33.3)	26 (43.3)	0.06
House without yard	10 (16.7)	4 (6.7)	
School attendance (%)			
Yes	30 (50)	24 (40)	0.01*
No	0 (0)	6 (10)	

**Notes:**

* *p*-value significant <0.05.

HGS, hand grip strength.

X^2^ and independent sample t-test.

A two-way ANOVA was conducted that compared the effect of gender and group on the average HGS and the KIDSCREEN-10 sum score. There was a statistically significant main effect of gender on the average HGS, *F* (1, 56) = 24.09, *p* < 0.001, main effect of gender on the KIDSCREEN-10 sum score, *F* (1, 56) = 8.66, *p* < 0.001, and statistically significant main effect of group on the KIDSCREEN-10 sum score, *F* (1, 56) = 17.64, *p* < 0.001. Table 2 presents the mean and standard deviation of average HGS and the KIDSCREEN-10 sum score in the sample divided by group and gender.

**Table 2:** The hand grip strength and KIDSCREEN-10 sum score of the sample divided by group and gender.

Variable	Gender	Group		Group × Gender
		Typically developed	Cerebral palsy	
HGS average	Boys	24.14 ± 9.1	19.76 ± 4.4	*F* = 24.09 *p* = 0.001*
	Girls	14.25 ± 9.1	11.71 ± 3.7	
KIDSCREEN-10 sum score	Boys	47.1 ± 2.18	44 ± 5.23	*F* = 8.66 *p* = 0.001*
	Girls	45.5 ± 5.22	38.6 ± 4.92	

**Notes:**

* *p*-value presented the significant difference between gender.

Data presented as Mean ± SD.

Two-way ANOVA.

HGS, hand grip strength

**Figure 2:**
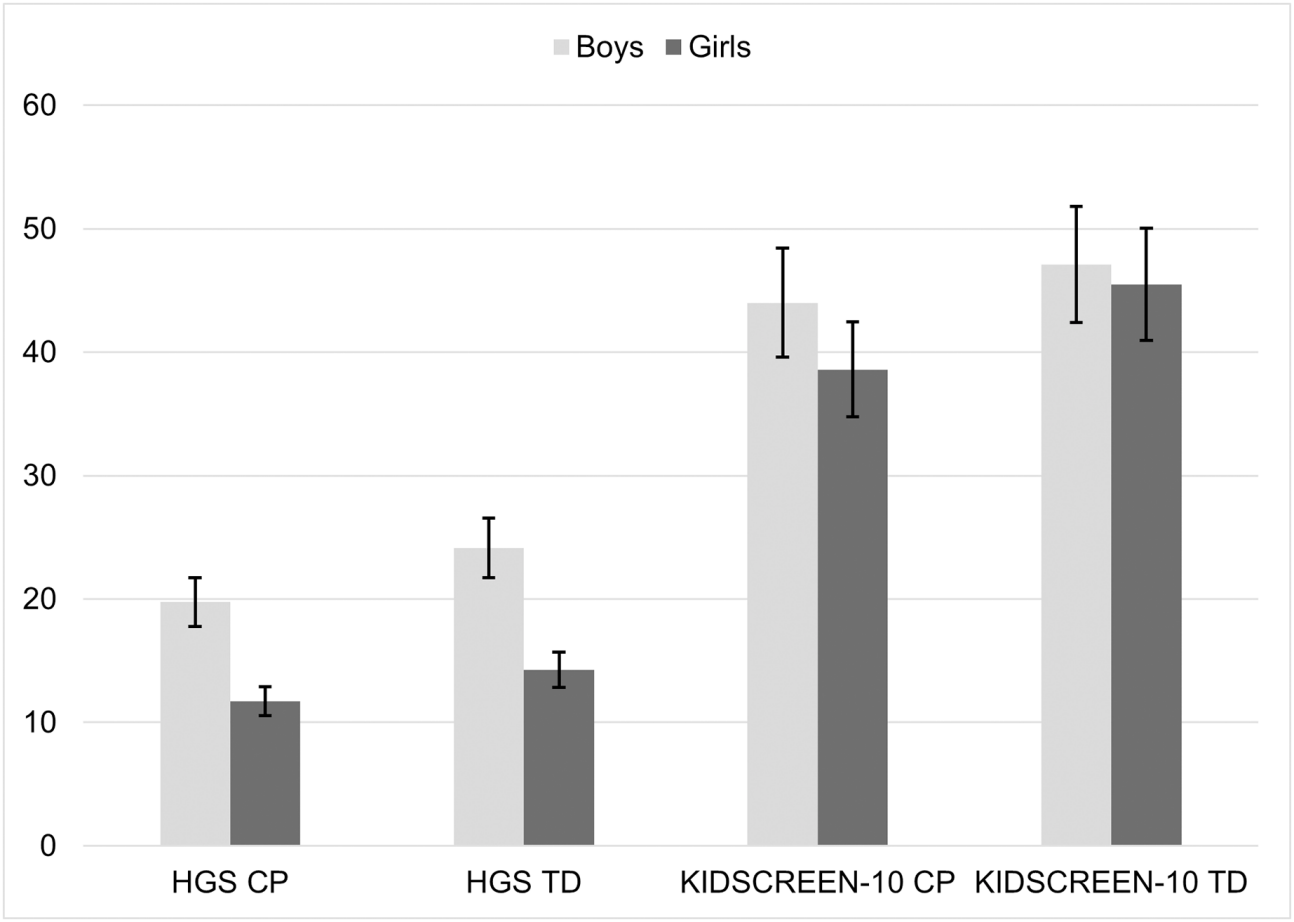
Difference between groups cerebral palsy (CP) *vs* typically developed (TD) and gender (boys *vs* girls) in average hand grip strength (HGS) and KIDSCREEN-10.

A *post hoc* comparison using the Tukey honestly significant difference (HSD) test revealed that the average HGS was lower in CP girls compared to TD girls, but not statistically significant (*p* = 0.71). The average HGS was lower in CP boys compared to TD boys, but not statistically significant (*p* = 0.36). A *post hoc* comparison using the Tukey HSD test revealed that the mean score of the KIDSCREEN-10 was significantly lower in CP girls compared to TD girls (*p* < 0.001) and was significantly lower in CP boys compared to TD boys (*p* < 0.001) (Fig. 2).

Table 3 presents the number and percentages of the KIDSCREEN-10 items reported by parent. Overall, CP patients had lower average on the majority of HRQoL items reported by parents. They have reported a lower score on general health status question (In general, how would your child rate her/his health?) at the end of the KIDSCREEN-10 questionnaire. A Mann–Whitney U test revealed a significant difference (*U* = 172, *p* < 0.001), between the CP group (4.07 ± 0.78) and TD group (4.93 ± 0.25).

**Table 3:** KIDSCREEN-10 items reported by parents divided by groups.

Item	Typically developed						Cerebral palsy					
	Answer category					Mean ± SD	Answer category					Mean ± SD
	1	2	3	4	5		1	2	3	4	5	
1. Has your child felt fit and well?	0	0	0	2 (3.3)	28 (46.7)	4.93 ± 0.25	4 (6.7)	0	8 (13.3)	12 (20)	6 (10)	3.53 ± 1.22
2. Has your child felt full of energy?	0	0	0	10 (16.7)	20 (33.3)	4.67 ± 0.47	0	0	8 (13.3)	10 (16.7)	12 (20)	4.13 ± 0.81
3. Has your child felt sad?	0	2 (3.3)	6 (10)	10 (16.7)	12 (20)	4.07 ± 0.94	0	2 (3.3)	12 (20)	12 (20)	4 (6.7)	3.60 ± 0.81
4. Has your child felt lonely?	0	0	6 (10)	2 (3.3)	22 (36.7)	4.53 ± 0.81	0	0	6 (10)	4 (6.7)	20 (33.3)	4.47 ± 0.81
5. Has your child had enough time for him/herself?	0	0	0	12 (20)	18 (30)	4.60 ± 0.49	2 (3.3)	2 (3.3)	4 (6.7)	2 (3.3)	20 (33.3)	4.20 ± 1.29
6. Has your child been able to do the things that he/she wants to do in his/her free time?	0	0	2 (3.3)	14 (23.3)	14 (23.3)	4.40 ± 0.62	2 (3.3)	0	4 (6.7)	20 (33.3)	4 (6.7)	3.80 ± 0.92
7. Has your child felt that his/her parent(s) treated him/her fairly?	0	0	2 (3.3)	2 (3.3)	26 (43.3)	4.80 ± 0.55	0	0	0	10 (16.7)	20 (33.3)	4.67 ± 0.47
8. Has your child had fun with his/her friends?	0	0	0	4 (6.7)	26 (43.3)	4.87 ± 0.34	0	2 (3.3)	0	12 (20)	16 (26.7)	4.40 ± 0.81
9. Has your child got on well at school?	0	0	2 (3.3)	6 (10)	22 (36.7)	4.67 ± 0.60	0	6 (10)	4 (6.7)	4 (6.7)	16 (26.7)	4 ± 1.23
10. Has your child been able to pay attention?	0	0	2 (3.3)	4 (6.7)	24 (40)	4.73 ± 0.58	0	0	6 (10)	8 (13.3)	16 (26.7)	4.33 ± 0.80
In general, how would your child rate her/his health?	0	0	0	2 (3.3)	28 (46.7)	4.93 ± 0.25	0	0	8 (13.3)	12 (20)	10 (16.7)	4.07 ± 0.78

**Notes:**

Data presented as number (percentage) and mean ± SD.

Answer categories item 1 and 9: (1) not at all; (2) slightly; (3) moderately; (4) very; and (5) extremely. All other items: (1) never; (2) seldom; (3) quite often; (4) very often; and (5) always.

A Spearman correlation was conducted to examine the relationship between HGS and KIDSCREEN-10 sum score among both groups. A significant correlation was recorded between HGS and the KIDSCREEN-10 sum score in CP group (*r* = 0.35, *p* = 0.03), and TD group (*r* = 0.56, *p* = 0.001). Table 4 summarizes the correlation coefficient between HGS and KIDSCREEN-10 sum score, age, and number of siblings.

**Table 4:** Correlation between hand grip strength and KIDSCREEN-10, age, and number of siblings divided by groups.

Variables	HGS typically developed		HGS cerebral palsy	
	r Spearman	*p*-value	r Spearman	*p*-value
KIDSCREEN-10 sum score	0.56	<0.001**	0.35	0.03*
Age	0.49	<0.001**	0.15	0.40
Number of siblings	0.33	0.07	0.14	0.45

**Notes:**

* Correlation is significant at the <0.05 level.

** Correlation is significant at the 0.01 level.

## Discussion

In this experimental cross-sectional study, we have investigated the relationship between HGS and HRQoL in children with CP compared to TD counterparts. Our results showed that HGS was lower in CP children, and girls had significantly lower HGS compared to boys in both groups, CP and TD children. Moreover, normalized HGS was greater in TD children compared to CP peers. Health-related quality of life was significantly lower in children with CP, with boys reporting higher HRQoL on the KIDSCREEN-10 questionnaire compared to girls. Our data showed that the higher KIDSCREEN-10 sum score is, the stronger HGS of children in both groups.

It is not a surprise that HGS in children with CP is lower than their TD counterparts due to the neurological impairments accompanying the brain damage in CP patients. Children with CP have a lower normalized HGS than TD children, suggesting that HGS is influenced by body mass, rather than muscle morphology or neural function. Children with higher body mass may have a mechanical advantage, allowing for stronger grip even if their muscle quality or neural function is not superior. Thus, differences in body mass can lead to variations in HGS between individuals (Rostamzadeh et al., 2021; Alkholy, El-Wahab & Elshennawy, 2017). Consequently, muscle strength evaluations should not rely solely on HGS measurements but also consider the child’s body weight to avoid misinterpretations. Additionally, alternative methods that assess muscle quality and neural function should be employed to gain a comprehensive understanding of a child’s physical development.

It has long been known that hand strength has an important role to play in activity participation and functional ability of children with CP (Brown et al., 1987). Musculoskeletal impairments, sarcopenia, and chronic pain are common in CP patients that make it difficult for them to perform activities of daily living (Stavsky et al., 2017; Heyn et al., 2019; Peterson, Gordon & Hurvitz, 2013). Research into the relationship between hand impairments and manual ability has indicated that motor functions such as grip strength, dexterity, and spasticity seem to be related to manual ability (Cans, 2000; Verschuren et al., 2007). In comparison with previously published normative data for TD children, our HGS data are slightly higher. Data collection procedures, sample size, and devices used may have contributed to this result (Alqahtani et al., 2023; Omar, Alghadir & Al Baker, 2015).

A variety of physiological factors, including muscle mass, hormonal differences, and varying levels of physical activity, contribute to gender differences in HGS, with boys typically showing greater strength than girls (Rostamzadeh et al., 2021; Górecki et al., 2024). Studies have found that boys experience a greater increase in muscle strength during puberty than girls, as these differences emerge early and become more pronounced during childhood (Al-Rahamneh et al., 2020; Wen et al., 2020). In children with CP, spasticity and muscle atrophy may differ by gender and may impact HGS. In line with our findings, previous studies have indicated that boys with CP may experience greater muscular development despite the condition, potentially resulting in greater grip strength than girls with CP, who might experience greater muscle weakness and coordination difficulties (Mehmood et al., 2023; Dekkers et al., 2020).

Although it is difficult to quantify QoL for a chronic and multifaceted condition such as CP, several studies have investigated this relationship (Dickinson et al., 2007; Bjornson et al., 2008; Du et al., 2010; Calley et al., 2012; Vles et al., 2015). In a recent systematic review published in 2019, eleven studies were included to compare the QoL of children and adolescents with CP compared to their TD peers. Overall, CP patients experienced limited ability to participate in physical activities such as self-care, exercise, and poor physical health (Makris, Dorstyn & Crettenden, 2021). There has also been a report of lowered self-esteem, poor mental health, and a negative impact on the parents’ own wellbeing as a result of their child’s disability. In two studies using the KIDSCREEN tool, parents have reported a higher QoL in relation to parent-child relationship and life at home. Further, they have reported a positive perception of the school environment, including engagement and learning (Dickinson et al., 2007; Wake, Salmon & Reddihough, 2003). As compared to our findings, we found similar results regarding limited QoL for children with CP compared to those with TD. Although these studies included a larger sample size, they also examined the differences between parent-reported and child-reported questionnaires. Parent-reported questionnaires are especially important when treating children with severe CP symptoms due to intellectual and/or communicative disabilities. It should be noted, however, that parent-reported questionnaires may introduce systematic bias as they are based on indirect cues and personal experience (Cummins, 2002).

Research indicates that gender can have an impact on the perception and experience of HRQoL in TD children (Chen et al., 2020). Different socialization patterns, expectations, and coping strategies among boys and girls can influence their mental and physical health. According to our study, boys with CP have higher HGS, suggesting that they have greater muscle strength and physical activity as compared to girls, which may be beneficial to their physical well-being. On the other hand, girls with CP may experience greater limitations in mobility and strength, which can lead to lower scores in HRQoL physical domains (Makris, Dorstyn & Crettenden, 2021; Sharawat & Panda, 2022). In accordance with our findings, recent studies have suggested that boys with CP may experience advantages in physical functioning and social support, leading to higher QoL scores compared to girls with CP (Park et al., 2016; Radsel, Osredkar & Neubauer, 2017; Tedla et al., 2024).

To our knowledge this is the first study to evaluate the relationship between the HGS and HRQoL in children with CP and their TD counterparts. Hand function is crucial to carry out day-to-day activities such as eating and dressing. Children with CP face these challenges frequently, which may negatively influence their activity level and social participation. We have presented a positive correlation between the KIDSCREEN-10 sum score and HGS in both children with CP and TD children. This correlation indicated that HGS influences different aspects of HRQoL, such as physical activity, depressive moods and emotions, social participation and leisure time, the quality of interaction between the child and their parents, and finally the cognitive ability perception. These findings serve as a baseline of future research which could further shed light into the influence of HGS on HRQoL of children with CP with different phenotypes, gross motor level, and manual ability level.

There are several limitations to this study. First, the cross-sectional study design does not prove a causal connection between HGS and HRQoL. A prospective study is necessary to examine the relationship between low HGS and associated variables in the future. Second, the limited sample size compared to previously published data suggests that caution should be exercised when interpreting these findings. Third, although the HGS tool used in the current study is easy to administer, cost-effective, and practical, its psychometric properties might concern some researchers. Future research should therefore use a more valid and reliable tool. Fourth, the KIDSCREEN-10 item is valid, reliable, and takes a few minutes to complete. However, its limited items, in contrast to the larger versions of the KIDSCREEN-52 and KIDSCREEN-27, prevented us from covering all HRQoL dimensions in this study. Finally, the generalizability of our conclusions to the wider CP population, such as those with GMFCS levels IV and V, is limited, hence caution should be exercised when interpreting the results.

## Conclusion

Overall, the results of this study indicate that HGS may significantly impact the HRQoL of children with CP. It was found that children with CP exhibited significantly lower levels of HGS than their TD peers. Disparities in this regard may have an adverse impact on their daily lives, negatively affecting their independence and participation in activities. A correlation between HGS and HRQoL points to the importance of improving strength in children with CP through interventions and directed rehabilitation programs. In order to improve the QoL for these children, it may be necessary to implement targeted interventions, such as occupational therapy or specialized exercises. Researchers must continue to explore the long-term impacts of interventions on QoL and HGS in both populations, ensuring that strategies are evidence-based and tailored to meet the individual needs of children with CP.

## Supplemental Information

10.7717/peerj.18679/supp-1 Supplemental Information 1 Raw data.

## Additional Information and Declarations

### Data Availability

The following information was supplied regarding data availability: The raw data is available at figshare: Alghadier, Mshari (2024). HGS and QoL in CP and TD children: figshare. Dataset. https://doi.org/10.6084/m9.figshare.27241548.v1.

## References

[ref-1] Al Salloum AA, El Mouzan MI, Al Omar AA, Al Herbish AS, Qurashi MM (2011). The prevalence of neurological disorders in Saudi children: a community-based study. Journal of Child Neurology.

[ref-2] Al-Jabri BA, Al-Amri AS, Jawhari AA, Sait RM, Talb RY (2022). Prevalence, types, and outcomes of cerebral palsy at a tertiary center in Jeddah, Saudi Arabia. Cureus.

[ref-3] Al-Rahamneh H, Aloran H, Alnader H, Al Ghafary N, Ismail W, Al Qarra S (2020). Reference values of hand-grip strength for 6-to 18-year-olds in Jordan. Journal of Exercise Physiology Online.

[ref-4] Alkholy WAS, El-Wahab MSE-D, Elshennawy S (2017). Hand grip strength in relation to anthropometric measures of school children: a cross sectional study. Annals of Medical and Health Science Research.

[ref-5] Allen D, Barnett F (2011). Reliability and validity of an electronic dynamometer for measuring grip strength. International Journal of Therapy and Rehabilitation.

[ref-6] Alqahtani BA, Alenazi AM, Elnaggar RK, Alshehri MM, Alhowimel A, Najmi AA, Alasraj M, Alghadeir M (2023). Normative values for hand grip and pinch strength for 6 to 18 year-olds in Saudi Arabia. BMC Musculoskeletal Disorders.

[ref-7] Altman DG (1990). Practical statistics for medical research.

[ref-8] American Society of Hand Therapists (1981). Clinical assessment recommendations.

[ref-9] Arnaud C, Ehlinger V, Delobel-Ayoub M, Klapouszczak D, Perra O, Hensey O, Neubauer D, Hollódy K, Virella D, Rackauskaite G, Greitane A, Himmelmann K, Ortibus E, Dakovic I, Andersen GL, Papavasiliou A, Sellier E, Platt MJ, Krägeloh-Mann I (2021). Trends in prevalence and severity of pre/perinatal cerebral palsy among children born preterm from 2004 to 2010: a SCPE collaboration study. Frontiers in Neurology.

[ref-10] Bax M, Goldstein M, Rosenbaum P, Leviton A, Paneth N, Dan B, Jacobsson B, Damiano D, Executive Committee for Definition of Cerebral Palsy (2005). Proposed definition and classification of cerebral palsy, April 2005. Developmental Medicine & Child Neurology.

[ref-11] Bingol H, Gunel MK, Asena Sel S, Burc E, Fidan H (2023). Validity and reliability of the Turkish version of the KIDSCREEN-27 for individuals with cerebral palsy. Perceptual and Motor Skills.

[ref-12] Bjornson KF, Belza B, Kartin D, Logsdon RG, McLaughlin J (2008). Self-reported health status and quality of life in youth with cerebral palsy and typically developing youth. Archives of Physical Medicine and Rehabilitation.

[ref-13] Bolger LE, Bolger LA, O’Neill C, Coughlan E, O’Brien W, Lacey S, Burns C, Bardid F (2021). Global levels of fundamental motor skills in children: a systematic review. Journal of Sports Sciences.

[ref-14] Braccialli LMP, Almeida VS, Sankako AN, Silva MZ, Braccialli AC, Carvalho SMR, Magalhães AT (2016). Translation and validation of the Brazilian version of the Cerebral Palsy Quality of Life Questionnaire for Children-child report. Journal of Pediatrics.

[ref-15] Brown JK, Van Rensburg F, Lakie GWM, Wrigh GW (1987). A neurological study of hand function of hemiplegic children. Developmental Medicine & Child Neurology.

[ref-16] Böling S, Tarja V, Helena M, Wivi F, Ilona A-R, Leena H (2013). Measuring quality of life of Finnish children with cerebral palsy. Journal of Pediatric Rehabilitation Medicine.

[ref-17] Calley A, Williams S, Reid S, Blair E, Valentine J, Girdler S, Elliott C (2012). A comparison of activity, participation and quality of life in children with and without spastic diplegia cerebral palsy. Disability and Rehabilitation.

[ref-18] Cans C (2000). Surveillance of cerebral palsy in Europe: a collaboration of cerebral palsy surveys and registers. Developmental Medicine & Child Neurology.

[ref-19] Cappellini G, Sylos-Labini F, Dewolf AH, Solopova IA, Morelli D, Lacquaniti F, Ivanenko Y (2020). Maturation of the locomotor circuitry in children with cerebral palsy. Frontiers in Bioengineering and Biotechnology.

[ref-20] Chen X, Cai Z, He J, Fan X (2020). Gender differences in life satisfaction among children and adolescents: a meta-analysis. Journal of Happiness Studies.

[ref-21] Colver A, Rapp M, Eisemann N, Ehlinger V, Thyen U, Dickinson HO, Parkes J, Parkinson K, Nystrand M, Fauconnier J, Marcelli M, Michelsen SI, Arnaud C (2015). Self-reported quality of life of adolescents with cerebral palsy: a cross-sectional and longitudinal analysis. The Lancet.

[ref-22] Cumberworth VL, Patel NN, Rogers W, Kenyon GS (2007). The maturation of balance in children. The Journal of Laryngology & Otology.

[ref-23] Cummins RA (2002). Proxy responding for subjective well-being: a review. International Review of Research in Mental Retardation.

[ref-24] Dapp LC, Gashaj V, Roebers CM (2021). Physical activity and motor skills in children: a differentiated approach. Psychology of Sport and Exercise.

[ref-25] Dekkers K, Janssen-Potten Y, Gordon AM, Speth L, Smeets R, Rameckers E (2020). Reliability of maximum isometric arm, grip and pinch strength measurements in children (7–12 years) with unilateral spastic cerebral palsy. Disability and Rehabilitation.

[ref-26] Dekkers KJFM, Rameckers EAA, Smeets RJEM, Gordon AM, Speth LAWM, Ferre CL, Janssen-Potten YJM (2020). Measuring upper extremity muscle strength in children with unilateral spastic cerebral palsy: a bilateral problem?. Physical Therapy.

[ref-27] Dickinson HO, Parkinson KN, Ravens-Sieberer U, Schirripa G, Thyen U, Arnaud C, Beckung E, Fauconnier J, McManus V, Michelsen SI, Parkes J, Colver AF (2007). Self-reported quality of life of 8–12-year-old children with cerebral palsy: a cross-sectional European study. The Lancet.

[ref-28] Du RY, McGrath C, Yiu CKY, King NM (2010). Health-and oral health-related quality of life among preschool children with cerebral palsy. Quality of Life Research.

[ref-30] Górecki M, Kazarców M, Protasewicz A, Czarnecki P, Romanowski L (2024). Population norms for hand grip and precision grip strengths in polish children and adolescents aged 3–19. Journal of Clinical Medicine.

[ref-31] Gąsior JS, Pawłowski M, Jeleń PJ, Rameckers EA, Williams CA, Makuch R, Werner B (2020). Test–retest reliability of handgrip strength measurement in children and preadolescents. International Journal of Environmental Research and Public Health.

[ref-32] Halaweh H (2020). Correlation between health-related quality of life and hand grip strength among older adults. Experimental Aging Research.

[ref-33] Heyn PC, Tagawa A, Pan Z, Reistetter T, Ng TKS, Lewis M, Carollo JJ (2023). The association between isometric strength and cognitive function in adults with cerebral palsy. Frontiers in Medicine.

[ref-34] Heyn PC, Tagawa A, Pan Z, Thomas S, Carollo JJ (2019). Prevalence of metabolic syndrome and cardiovascular disease risk factors in adults with cerebral palsy. Developmental Medicine & Child Neurology.

[ref-35] Im Y, Cho Y, Kim D (2019). Family management style as a mediator between parenting stress and quality of life of children with epilepsy. Journal of Pediatric Nursing.

[ref-36] Jonsson U, Eek MN, Sunnerhagen KS, Himmelmann K (2019). Cerebral palsy prevalence, subtypes, and associated impairments: a population-based comparison study of adults and children. Developmental Medicine & Child Neurology.

[ref-37] Kang SY, Lim J, Park HS (2018). Relationship between low handgrip strength and quality of life in Korean men and women. Quality of Life Research.

[ref-38] Lorentzen J, Willerslev-Olsen M, Hüche Larsen H, Farmer SF, Nielsen JB (2019). Maturation of feedforward toe walking motor program is impaired in children with cerebral palsy. Brain.

[ref-39] Maier M, Stoltenburg C, Sarpong-Bengelsdorf A, Lebek S (2022). Validity and reliability of the German version of the CP QOL-child and CP QOL-teen questionnaire. Neuropediatrics.

[ref-40] Makris T, Dorstyn D, Crettenden A (2021). Quality of life in children and adolescents with cerebral palsy: a systematic review with meta-analysis. Disability and Rehabilitation.

[ref-41] Mandanka N, Diwan S (2020). Intra rater and inter rater reliability of hand dynamometer and pinch gauge in children with spastic cerebral palsy. Indian Journal of Physiotherapy and Occupational Therapy.

[ref-42] Mc Manus V, Corcoran P, Perry IJ (2008). Participation in everyday activities and quality of life in pre-teenage children living with cerebral palsy in South West Ireland. BMC Pediatrics.

[ref-43] Mehmood A, Ammad HU, Afzal MF, Amjad A, Hasnat A, Saeed A (2023). Assessment of handgrip strength in spastic diplegic cerebral palsy children. Journal Riphah College of Rehabilitation Sciences.

[ref-44] Michael-Asalu A, Taylor G, Campbell H, Lelea L-L, Kirby RS (2019). Cerebral palsy: diagnosis, epidemiology, genetics, and clinical update. Advances in Pediatrics.

[ref-45] Milićević M (2023). Functional and environmental predictors of health-related quality of life of school-age children with cerebral palsy: a cross-sectional study of caregiver perspectives. Child: Care, Health and Development.

[ref-46] Musalek C, Kirchengast S (2017). Grip strength as an indicator of health-related quality of life in old age—a pilot study. International Journal of Environmental Research and Public Health.

[ref-47] Odding E, Roebroeck ME, Stam HJ (2006). The epidemiology of cerebral palsy: incidence, impairments and risk factors. Disability and Rehabilitation.

[ref-48] Omar MTA, Alghadir A, Al Baker S (2015). Norms for hand grip strength in children aged 6–12 years in Saudi Arabia. Developmental Neurorehabilitation.

[ref-49] Ortega FB, Cadenas-Sánchez C, Sánchez-Delgado G, Mora-González J, Martínez-Téllez B, Artero EG, Castro-Piñero J, Labayen I, Chillón P, Löf M, Ruiz JR (2015). Systematic review and proposal of a field-based physical fitness-test battery in preschool children: the PREFIT battery. Sports Medicine.

[ref-50] Park SK, Yang DJ, Heo JW, Kim JH, Park SH, Uhm YH (2016). Study on the quality of life of children with cerebral palsy. Journal of Physical Therapy Science.

[ref-51] Patel DR, Neelakantan M, Pandher K, Merrick J (2020). Cerebral palsy in children: a clinical overview. Translational Pediatrics.

[ref-52] Paul S, Nahar A, Bhagawati M, Kunwar AJ (2022). A review on recent advances of cerebral palsy. Oxidative Medicine and Cellular Longevity.

[ref-53] Peolsson A, Hedlund R, Öberg B (2001). Intra-and inter-tester reliability and reference values for hand strength. Journal of Rehabilitation Medicine.

[ref-54] Peterson MD, Gordon PM, Hurvitz EA (2013). Chronic disease risk among adults with cerebral palsy: the role of premature sarcopoenia, obesity and sedentary behaviour. Obesity Reviews.

[ref-55] Power R, Galea C, Muhit M, Heanoy E, Karim T, Badawi N, Khandaker G (2020). What predicts the proxy-reported health-related quality of life of adolescents with cerebral palsy in Bangladesh?. BMC Public Health.

[ref-56] Radsel A, Osredkar D, Neubauer D (2017). Health-related quality of life in children and adolescents with cerebral palsy. Slovenian Journal of Public Health.

[ref-57] Rapp M, Eisemann N, Arnaud C, Ehlinger V, Fauconnier J, Marcelli M, Michelsen SI, Nystrand M, Colver A, Thyen U (2017). Predictors of parent-reported quality of life of adolescents with cerebral palsy: a longitudinal study. Research in Developmental Disabilities.

[ref-58] Ravens-Sieberer U, Auquier P, Erhart M, Gosch A, Rajmil L, Bruil J, Power M, Duer W, Cloetta B, Czemy L, Mazur J, Czimbalmos A, Tountas Y, Hagquist C, Kilroe J, the European KIDSCREEN Group (2007). The KIDSCREEN-27 quality of life measure for children and adolescents: psychometric results from a cross-cultural survey in 13 European countries. Quality of Life Research.

[ref-59] Ravens-Sieberer U, Erhart M, Rajmil L, Herdman M, Auquier P, Bruil J, Power M, Duer W, Abel T, Czemy L, Mazur J, Czimbalmos A, Tountas Y, Hagquist C, Kilroe J, the European KIDSCREEN Group (2010). Reliability, construct and criterion validity of the KIDSCREEN-10 score: a short measure for children and adolescents’ well-being and health-related quality of life. Quality of Life Research.

[ref-60] Ravens-Sieberer U, Gosch A, Rajmil L, Erhart M, Bruil J, Duer W, Auquier P, Power M, Abel T, Czemy L, Mazur J, Czimbalmos A, Tountas Y, Hagquist C, Kilroe J, European KIDSCREEN Group (2005). KIDSCREEN-52 quality-of-life measure for children and adolescents. Expert Review of Pharmacoeconomics & Outcomes Research.

[ref-61] Ravens-Sieberer U, Gosch A, Rajmil L, Erhart M, Bruil J, Power M, Duer W, Auquier P, Cloetta B, Czemy L, Mazur J, Czimbalmos A, Tountas Y, Hagquist C, Kilroe J, the KIDSCREEN Group (2008). The KIDSCREEN-52 quality of life measure for children and adolescents: psychometric results from a cross-cultural survey in 13 European countries. Value in Health.

[ref-100] R Core Team (2020). R: a language and environment for statistical computing.

[ref-62] Robertson CMT, Ricci MF, O’Grady K, Oskoui M, Goez H, Yager JY, Andersen JC (2017). Prevalence estimate of cerebral palsy in Northern Alberta: births, 2008–2010. Canadian Journal of Neurological Sciences.

[ref-63] Robitail S, Ravens-Sieberer U, Simeoni M-C, Rajmil L, Bruil J, Power M, Duer W, Cloetta B, Czemy L, Mazur J, Czimbalmos A, Tountas Y, Hagquist C, Kilroe J, Auquier P, the KIDSCREEN Group (2007). Testing the structural and cross-cultural validity of the KIDSCREEN-27 quality of life questionnaire. Quality of Life Research.

[ref-64] Rosenbaum PL, Walter SD, Hanna SE, Palisano RJ, Russell DJ, Raina P, Wood E, Bartlett DJ, Galuppi BE (2002). Prognosis for gross motor function in cerebral palsy: creation of motor development curves. The Journal of the American Medical Association.

[ref-65] Rostamzadeh S, Saremi M, Abouhossein A, Vosoughi S, Molenbroek JFM (2021). Normative data for handgrip strength in Iranian healthy children and adolescents aged 7–18 years: comparison with international norms. Italian Journal of Pediatrics.

[ref-66] Rostamzadeh S, Saremi M, Vosoughi S, Bradtmiller B, Janani L, Farshad AA, Taheri F (2021). Analysis of hand-forearm anthropometric components in assessing handgrip and pinch strengths of school-aged children and adolescents: a partial least squares (PLS) approach. BMC Pediatrics.

[ref-67] Savva C, Giakas G, Efstathiou M, Karagiannis C (2014). Test–retest reliability of handgrip strength measurement using a hydraulic hand dynamometer in patients with cervical radiculopathy. Journal of Manipulative and Physiological Therapeutics.

[ref-68] Schreuders TAR, Roebroeck ME, Goumans J, van Nieuwenhuijzen JF, Stijnen TH, Stam HJ (2003). Measurement error in grip and pinch force measurements in patients with hand injuries. Physical Therapy.

[ref-69] Sharawat IK, Panda PK (2022). Quality of life and its association with level of functioning in young children with cerebral palsy. Neuropediatrics.

[ref-70] Shikako-Thomas K, Dahan-Oliel N, Shevell M, Law M, Birnbaum R, Rosenbaum P, Poulin C, Majnemer A (2012). Play and be happy? Leisure participation and quality of life in school-aged children with cerebral palsy. International Journal of Pediatrics.

[ref-71] Solans M, Pane S, Estrada M, Serra-Sutton V, Berra S, Herdman M, Alonso J, Rajmil L (2008). Health-related quality of life measurement in children and adolescents: a systematic review of generic and disease-specific instruments. Value in Health.

[ref-72] Stavsky M, Mor O, Mastrolia SA, Greenbaum S, Than NG, Erez O (2017). Cerebral palsy—trends in epidemiology and recent development in prenatal mechanisms of disease, treatment, and prevention. Frontiers in Pediatrics.

[ref-73] Tedla JS, Sangadala DR, Asiri F, Alshahrani MS, Alkhamis BA, Reddy RS, Gular K, Alamri AM, Alwadei ARS, Mukherjee D (2024). Quality of life among children with cerebral palsy in the Kingdom of Saudi Arabia and various factors influencing it: a cross-sectional study. Journal of Disability Research.

[ref-74] The KIDSCREEN Group Europe (2006). The KIDSCREEN questionnaires: quality of life questionnaires for children and adolescents.

[ref-29] The WHOQOL Group (1995). The World Health Organization quality of life assessment (WHOQOL): position paper from the World Health Organization. Social Science & Medicine.

[ref-75] Touyama M, Touyama J, Toyokawa S, Kobayashi Y (2016). Trends in the prevalence of cerebral palsy in children born between 1988 and 2007 in Okinawa, Japan. Brain and Development.

[ref-76] Trossman PB, Li P-W (1989). The effect of the duration of intertrial rest periods on isometric grip strength performance in young adults. The Occupational Therapy Journal of Research.

[ref-77] Tóth R (2017). Improvement of fine motor skills in cerebral paretic patients. Különleges Bánásmód-Interdiszciplináris Folyóirat.

[ref-78] Van der Fels IMJ, Te Wierike SCM, Hartman E, Elferink-Gemser MT, Smith J, Visscher C (2015). The relationship between motor skills and cognitive skills in 4–16 year old typically developing children: a systematic review. Journal of Science and Medicine in Sport.

[ref-79] Verschuren O, Ketelaar M, Gorter JW, Helders PJM, Uiterwaal CSPM, Takken T (2007). Exercise training program in children and adolescents with cerebral palsy: a randomized controlled trial. Archives of Pediatrics and Adolescent Medicine.

[ref-80] Vitrikas K, Dalton H, Breish D (2020). Cerebral palsy: an overview. American Family Physician.

[ref-81] Vles GF, Hendriksen RGF, Hendriksen JGM, van Raak EPM, Soudant D, Vles JSH, Gavilanes AWD (2015). Quality of life of children with cerebral palsy: a cross-sectional KIDSCREEN study in the southern part of the Netherlands. CNS & Neurological Disorders-Drug Targets.

[ref-82] Wake M, Salmon L, Reddihough D (2003). Health status of Australian children with mild to severe cerebral palsy: cross-sectional survey using the Child Health Questionnaire. Developmental Medicine & Child Neurology.

[ref-83] Wen J, Wang J, Xu Q, Wei Y, Zhang L, Ou J, Hong Q, Ji C, Chi X, Tong M (2020). Hand anthropometry and its relation to grip/pinch strength in children aged 5 to 13 years. Journal of International Medical Research.

[ref-84] Wong SL (2016). Grip strength reference values for Canadians aged 6 to 79: Canadian Health Measures Survey, 2007 to 2013. Health Reports.

